# Phytoconstituents of *Androstachys johnsonii* Prain Prevent Reactive Oxygen Species Production and Regulate the Expression of Inflammatory Mediators in LPS-Stimulated RAW 264.7 Macrophages

**DOI:** 10.3390/antiox13040401

**Published:** 2024-03-27

**Authors:** Emmanuel Mfotie Njoya, Gaetan T. Tabakam, Chika I. Chukwuma, Samson S. Mashele, Tshepiso J. Makhafola

**Affiliations:** Centre for Quality of Health and Living, Faculty of Health and Environmental Sciences, Central University of Technology, Bloemfontein 9300, Free State, South Africa; tgaetan@cut.ac.za (G.T.T.); cchukwuma@cut.ac.za (C.I.C.); smashele@cut.ac.za (S.S.M.)

**Keywords:** *Androstachys johnsonii*, reactive oxygen species, pro-inflammatory mediators, flavonoids, phenolics, macrophages

## Abstract

According to a survey, the medicinal use of *Androstachys johnsonii* Prain is kept secret by traditional healers. Considering that inflammation and oxidative stress are major risk factors for the progression of various chronic diseases and disorders, we resolved to investigate the antioxidant and anti-inflammatory potentials of *A. johnsonii* using in vitro and cell-based assays. The antioxidant activity of *A. johnsonii* hydroethanolic leaf extract (AJHLE) was evaluated using the ABTS, DPPH, and FRAP assays. Its cytotoxic effect was assessed on RAW 264.7 macrophages using an MTT assay. Then, its anti-inflammatory effect was evaluated by measuring the NO production and 15-LOX inhibitory activities. Moreover, its preventive effect on ROS production and its regulatory effect on the expression of pro-inflammatory mediators such as IL-1β, IL-10, TNF-α, and COX-2 were determined using established methods. AJHLE strongly inhibited radicals such as ABTS^•+^, DPPH^•^, and Fe^3+^-TPTZ with IC_50_ values of 9.07 µg/mL, 8.53 µg/mL, and 79.09 µg/mL, respectively. Additionally, AJHLE induced a significant (*p* < 0.05) cytotoxic effect at 100 µg/mL, and when tested at non-cytotoxic concentrations, it inhibited NO and ROS production in LPS-stimulated RAW 264.7 macrophages in a concentration-dependent manner. Furthermore, AJHLE showed that its anti-inflammatory action occurs via the inhibition of 15-LOX activity, the downregulation of COX-2, TNF-α, and IL-1β expression, and the upregulation of IL-10 expression. Finally, chemical investigation showed that AJHLE contains significant amounts of procyanidin, epicatechin, rutin, and syringic acid which support its antioxidant and anti-inflammatory activities. These findings suggest that *A. johnsonii* is a potential source of therapeutic agents against oxidative stress and inflammatory-related diseases.

## 1. Introduction

Inflammation occurs within the body as an immune system’s response to harmful stimuli, such as pathogenic infections; exposure to radiation, allergens, and toxic chemicals; and other injuries [1]. In this regard, inflammation is a defensive mechanism that is essential to health. In fact, after exposure to pathogenic infections, the immune system reacts by activating different inflammatory cells, including macrophages, that release free radicals such as reactive oxygen species (ROS) and reactive nitrogen species (RNS), inducing damage to pathogens. Additionally, these damaging effects mobilize a cascade of interactions among inflammatory cells, thereby activating the production of prostaglandin E_2_ (PGE_2_), pro-inflammatory cytokines (e.g., tumor necrosis factor α (TNF-α), interleukins (IL)-1β, and IL-6), and enzymes such as cyclo-oxygenase-2 (COX-2) and inducible nitric oxide (iNOS) [2,3]. On the other hand, the role of 12/15-lipoxygenase (12/15-LOX) has been recognized in several inflammatory-related diseases. This is because 12/15-LOX is an oxidative enzyme that catalyzes the insertion of oxygen (O_2_) into poly-unsaturated fatty acids (PUFAs) such as arachidonic acid and linoleic acid, thereby generating pro-inflammatory lipid mediators known as leukotrienes [4]. Taken together, an excessive release of pro-inflammatory mediators will lead to chronic inflammation, which is recognized as a major risk factor for **the** progression of various chronic diseases and disorders. Lipopolysaccharide (LPS), a major constituent of Gram-negative bacteria’s cell wall and a potent stimulator of macrophages, induces inflammatory response by binding to Toll-like receptor 4 (TLR-4), which activates the release of inflammatory mediators along with downstream signaling pathways such as nuclear factor κB (NF-κB) and mitogen-activating protein kinase (MAPK) pathways [5]. Hence, we will take advantage of this experimental model to study the anti-inflammatory potential of *A. johnsonii* and draw further perspectives towards the medicinal use of this plant against chronic diseases.

Nonsteroidal anti-inflammatory drugs (NSAIDs) are commonly used for the management of pain, fever, and inflammatory conditions which are mostly associated in the progression of various chronic diseases [6]. However, the majority of NSAIDs also cause serious side effects, including gastrointestinal and renal complications [7]. Thus, the development of potent drugs against inflammatory conditions, and the emergence of drug resistance, have prompted research focusing on medicinal plants as a potential source of anti-inflammatory compounds with fewer side effects. In fact, medicinal plants are now universally recognized as alternative solutions to critical human health problems, and they also contribute to a growing economy and creation of jobs in developing countries [8,9]. Additionally, bioactive compounds of plant origin have inspired the development of many therapeutic drugs for the treatment of diseases around the world. South Africa has about 24,000 plant species with more than 50% being endemic plant species [10], and approximately 10% of the total flora has been recorded as being used for traditional medicine [11]. Although research carried out on plants is published every year, only a very small percentage (5–15%) of plants have been phytochemically and pharmacologically examined for their potential as sources of drugs [12,13]. Due to these facts, South African plants represent a great source of novel bioactive compounds for drug development. *Androstachys johnsonii* Prain was previously categorized as part of the Euphorbiaceae family but was later reclassified in the Picrodendraceae family (https://www.ipni.org/ (accessed on 12 February 2023)). *A. johnsonii* is an evergreen tree with a maximum height of about 15 m, and is widely used because of the strength, durability, and hardness of its wood [14]. This plant species remarkably keeps its leaves throughout the year, thus providing forage and shade to wildlife when other trees are leafless [15]. Additionally, *A. johnsonii* is known to secrete secondary metabolites that suppress the establishment and growth of other species under its canopy [16]. Very limited studies have reported the usage of this plant in folk medicine, as its medicinal uses are kept secret by traditional healers. However, an ethnobotanical survey has reported that the leaves, bark, and roots of *A. johnsonii* are used for stomach and sexual problems by local communities in Venda and Limpopo in South Africa [17] while only its root extract is being used as an aphrodisiac in Zimbabwe [18,19]. In this regard, research conducted so far on this plant species has revealed its antifungal [20] and antibacterial properties [17]. In another study, *A. johnsonii* methanolic leaf extract showed no mutagenic and antimutagenic effects against *Salmonella typhimurium* bacterial strains (TA98 and TA100) in the Ames test, thus indicating a lack of toxicity, which is a positive step towards the safe use of this plant species in traditional medicine [21]. To the best of our knowledge, no study has been carried out to investigate the potential of *A. johnsonii* against chronic diseases. Since inflammation and oxidative stress are major risk factors for the progression of various chronic diseases, the current study aimed to determine the antioxidant and anti-inflammatory potentials of *A. johnsonii* using in vitro and cell-based assays. Additionally, a chemical investigation was conducted to determine the phytochemicals that might be responsible for the biological properties of this plant species.

## 2. Material and Methods

### 2.1. Plant Material and Extraction Process

Fresh leaves of *A. johnsonii* were collected at Walter Sisulu National Botanical Garden, Gauteng (South Africa), and the herbarium specimen was identified at the HGWJ Schweickerdt Herbarium (PRU), University of Pretoria, under the number PRU 114701. The leaves were dried at room temperature in a well-ventilated room, and the dried leaves were ground to a fine powder. The leaf’s powder of *A. johnsonii* (50 g) was extracted in 500 mL of the mixture ethanol/water (80:20) at room temperature by maceration with continuous shaking for 48 h. The mixture was filtered through Whatman No.1 filter paper into pre-weighed beakers and the filtrate obtained was concentrated to dryness under a stream of cold air to obtain a residue which constituted the crude extract. The beakers were re-weighed to determine the quantity extracted, and the percentage yield of extraction was calculated (Table 1). The *A. johnsonii* hydroethanolic leaf extract (AJHLE) was stored in a cold room (2–4 °C) until use.

### 2.2. Antioxidant Assays

#### 2.2.1. ABTS^•+^ Radical Scavenging Assay

The method described by Miller et al. [22] and modified by Re et al. [23] was used for the determination of the ABTS^•+^ radical scavenging capacity of AJLHE. Precisely, the ABTS^•+^ radical cation was produced by mixing 7 mM ABTS solution and 2.45 mM potassium persulfate both dissolved in methanol, and the mixture was kept at room temperature (25 °C) for at least 16 h. The absorbance of the ABTS^•+^ radical produced was calibrated to 0.70 ± 0.02 at 734 nm on a SpectraMax iD3 multi-mode microplate reader (Molecular Devices, San Jose, CA, USA). On a 96-well microtiter plate, 40 µL of AJHLE (1 mg/mL) was serially diluted with methanol, followed by the addition of 160 µL (ABTS^•+^) radical cation, and the plate was incubated in the dark at room temperature for 5 min. The absorbance values were recorded against the blank (extract plus methanol without ABTS^•+^) at 734 nm a on a SpectraMax iD3 multi-mode microplate reader. Methanol plus ABTS^•+^ radical cation was used as negative control while ascorbic acid and quercetin (0–200 µg/mL) were used as positive controls. The percentage of ABTS^•+^ radical scavenging capacity was calculated at each concentration using Formula (1) below, and the inhibitory concentration (IC_50_) values were determined by plotting a non-linear curve of percentage ABTS^•+^ scavenging capacity versus a logarithm of tested concentrations.

Scavenging capacity (%) = [(A_0_ − A_1_)/A_0_] × 100 (1)

where A_0_ is the absorbance of the negative control and A_1_ is the absorbance of the extract with the ABTS^•+^ radical cation.

#### 2.2.2. DPPH^•^ Radical Scavenging Assay

The method described by Brand-Williams et al. [24] was applied for the determination of the DPPH^•^ scavenging capacity of AJHLE. On a 96-well microtiter plate, 40 µL of AJHLE (1 mg/mL) was serially diluted with methanol, followed by the addition of 160 µL of DPPH^•^ solution (25 µg/mL). The plates were incubated at room temperature (25 °C) in the dark for 30 min, and the absorbance values were measured against a blank (extract plus methanol without DPPH^•^) at 517 nm on a SpectraMax iD3 multi-mode microplate reader (Molecular Devices, USA). Ascorbic acid and quercetin (0–200 µg/mL) were used as positive controls while methanol plus DPPH^•^ was used as a negative control. The DPPH^•^ scavenging capacity was determined at tested concentrations according to Formula (1) above, and the IC_50_ values were also determined as mentioned above.

#### 2.2.3. Ferric Reducing Antioxidant Power (FRAP) Assay

The FRAP assay was performed using the method described by Benzie and Strain [25] to determine the antioxidant power in a redox-linked colorimetric reaction, where ferric-tripyridyltriazine (Fe^3+^-TPTZ) is reduced at a low pH to an intense blue-color ferrous-tripyridyltriazine complex (Fe^2+^-TPTZ) [26]. On a 96-well plate, 50 µL of AJLHE (1.56–200 µg/mL) or 200 µg/mL of gallic acid (Merck, South Africa) was incubated with 50 µL of 1% potassium ferricyanide (prepared in 0.2 M sodium phosphate buffer, pH 6.6) for 30 min at 50 °C. Thereafter, 50 µL of 10% trichloroacetic acid (TCA), 40 µL of distilled water, and 10 µL of 0.1% ferric chloride were added, respectively. The absorbance values were recorded against a blank (extract plus sodium phosphate buffer) at 700 nm on a SpectraMax iD3 multi-mode microplate reader (Molecular Devices, USA), and the FRAP activity was expressed as a percentage inhibition of 200 µg/mL gallic acid. Ascorbic acid (0–200 µg/mL) was used as a positive control.

### 2.3. Anti-Inflammatory Assays

#### 2.3.1. Cell Culture of Murine RAW 264.7 Macrophages and Cell Viability Assay

Murine RAW 264.7 macrophages were obtained from the American Type Culture Collection (ATCC^®^ TIB-71™) (Rockville, MD, USA), and they were grown in Roswell Park Memorial Institute (RPMI) 1640 medium (Cytiva Hyclone, Logan, UT, USA), supplemented with 10% fetal bovine serum (FBS) and 1% penicillin/streptomycin (P/S) in a CO_2_ incubator (Nϋve, Ankara, Turkey) under standard cell-culture conditions (37 °C in a humidified environment with 95% air and 5% CO_2_). At 70–80% confluency, the cells were trypsinised with 0.25% trypsin/EDTA (Cytiva Hyclone, USA) and split at a ratio of 1:5 for further passaging. The cell viability was checked with trypan blue 0.4% (Cytiva Hyclone, USA) on an automat cell counter (NanoEntek, Seoul, South Korea), and only suspensions with cell viabilities higher than 90% were used in our experiments.

The 3-(4,5-dimethythiazol-2-yl)-2,5-diphenyl tetrazolium bromide (MTT) assay [27] was used to evaluate the cell viability. In fact, murine RAW 264.7 cells were seeded at a density of 2 × 10^4^ cells per well on a 96-well microtiter plate, and the plate was incubated overnight under standard cell-culture conditions to allow for attachment. Thereafter, the cells were treated with AJHLE (3.125–100 µg/mL) and DMSO (0.5%) used as negative control, and the plate was incubated for 24 h under standard cell-culture conditions. After exposure, the culture medium containing the extract was discarded and replaced by fresh medium (200 µL) with 30 µL of thiazolyl blue tetrazolium bromide (5 mg/mL) (Melford, Ipswich, UK) dissolved in phosphate-buffered saline (PBS). After 4 h of incubation under standard cell-culture conditions, the culture medium was gently aspirated, and the formazan crystals were dissolved in 100 µL of DMSO and kept in the dark for 15 min at room temperature. The absorbance was measured spectrophotometrically at 570 nm on a SpectraMax iD3 multi-mode microplate reader (Molecular Devices, San Jose, CA, USA).

#### 2.3.2. Nitric Oxide Production Inhibitory Assay

RAW 264.7 cells were seeded at a density of 2 × 10^4^ cells per well on a 96-well microtiter plate, and the plate was incubated overnight under standard cell-culture conditions to allow attachment. Then, the cells were pre-treated for 2 h with different concentrations of AJHLE and quercetin (3.125–100 µg/mL) dissolved in DMSO, and further diluted in culture medium. Thereafter, lipopolysaccharide (LPS) (Merck, Darmstadt, Germany) was added to obtain a final concentration of 1 µg/mL, and further incubated for 24 h under standard cell-culture conditions. The cells treated with DMSO at 0.5% with LPS were considered as the negative control. Nitric oxide (NO) released by murine RAW 264.7 macrophages was measured using the Griess reagent (Sigma Aldrich, Hamburg, Germany) [28]. After exposure, 100 µL of cell supernatant from each well was transferred into a new 96-well microtiter plate, and an equal volume of Griess reagent was added. The plates were kept in the dark at room temperature (25 °C) for 15 min, and the absorbance values were measured at 540 nm on a SpectraMax iD3 multi-mode microplate reader (Molecular Devices, USA). The quantity of nitrite was calculated from a standard curve (y = 0.0029x + 0.0114; R^2^ = 0.994) generated with sodium nitrite (0–100 µM). The percentage of NO inhibition was determined based on the ability of each tested sample to inhibit nitric oxide production by LPS-stimulated RAW 264.7 macrophages compared with the negative control (cells treated with DMSO at 0.5% and LPS without extract).

#### 2.3.3. Quantification of Cyclo-Oxygenase (COX-2) and Cytokine Expression in LPS-Stimulated RAW 264.7 Cells

This was performed with available commercial kits using the enzyme-linked immune sorbent assay (ELISA) technique. In fact, murine RAW 264.7 cells were seeded at a density of 2 × 10^5^ cells per well into 12-well plates, and they were allowed to attach overnight. These cells were pre-treated with DMSO 0.5% (negative control), quercetin at 25 µM (positive control), and AJHLE at different concentrations (12.5, 25, and 50 µg/mL) for 2 h, followed by the addition of LPS (200 ng/mL) for 24 h under standard cell-culture conditions. Thereafter, treated cells were washed twice with pre-cooled phosphate-buffered saline (PBS) (Cytiva Hyclone, Logan, UT, USA) and dissociated using trypsin-EDTA (Cytiva Hyclone, Logan, UT, USA). The cell lysates were obtained in PBS containing EDTA-free Pierce Protease and Phosphatase-inhibitor Tablets (ThermoFisher Scientific, Lenexa, KS, USA) by repeating freeze–thaw cycles in liquid nitrogen and a water bath (37 °C). After centrifugation at 10,000 rpm at 4 °C, the cell supernatants were collected and used for the quantification of cyclooxygenase-2 (COX-2), interleukins (IL-1β and IL-10), and tumor necrosis factor (TNF-α) concentrations using Elabscience^®^ Mouse COX-2, IL-1β, IL-10, and TNF-α ELISA kits (Elabscience Biotechnology Inc., Houston, TX, USA) according to the manufacturer’s instructions.

#### 2.3.4. Measurement of Intracellular Reactive Oxygen Species (ROS) Levels

RAW 264.7 cells (1 × 10^4^), seeded on black/clear bottom 96-well-microtiter plate, were pre-treated with AJHLE at non-toxic concentrations (12.5, 25, and 50 µg/mL) for 2 h and then exposed or not to LPS (200 ng/mL) for 24 h under standard culture conditions. At the end of the experiment, the fluorescent probe 2′,7′dichlorodihydrofluorescein diacetate (DCFH-DA) (Sigma-Aldrich, Hamburg, Germany), was used to evaluate the intracellular ROS levels [29]. Precisely, after washing the cells with PBS, fresh culture medium without FBS, and containing 10 µM of DCFH-DA, was added and further incubated under standard culture conditions for 30 min. After this period, the fluorescent probe was replaced with PBS, and the cell fluorescence was measured at 485 nm (excitation) and 535 nm (emission) with a SpectraMax iD3 multi-mode microplate reader (Molecular Devices, USA). Intracellular ROS levels were expressed as percentages of negative control (cells treated with DMSO 0.5% without LPS). Images were captured using a Flexacam C1 camera (Leica Microsystems GmbH, Wetzlar, Germany) connected to a fluorescence microscope (Leica Microsystems GmbH, Wetzlar, Germany) using an excitation/emission filter (480/535 nm) on 40X objective.

#### 2.3.5. Soybean 15-Lipoxygenase (15-LOX) Enzyme Inhibitory Assay

The assay was performed according to the method developed by Pinto et al. [30] with slight modifications to the 96-well-microtiter-plate format. This method is based on the formation of the complex Fe^3+^/xylenol orange with a maximal absorption at 560 nm. The soyabean 15-LOX (Merck, Germany) (40 µL, 200 UI/mL) was incubated at 25 °C for 5 min with 20 µL of AJHLE at different concentrations (0.78 to 100 µg/mL). DMSO at 5% (*v/v*) was used as a negative control while gallic acid (0.78 to 100 µg/mL) was used as a standard inhibitor of 15-LOX. Thereafter, 40 µL of linoleic acid (final concentration, 140 μM) prepared in Tris-HCl buffer (50 mM, pH 7.4) was added, and the plate was incubated at 25 °C for 20 min in the dark. The assay was terminated by adding 100 µL of FOX reagent [sulfuric acid (30 mM), xylenol orange (100 μM), ferrous II) sulfate (100 μM), and dissolved in methanol/water (9:1)]. The plate was further incubated at 25 °C for 30 min in the dark, and the absorbance was read at 560 nm on a SpectraMax iD3 multi-mode microplate reader (Molecular Devices, USA). The blanks were made in the same way as tested samples except that the substrate was added after the FOX reagent. The 15-LOX inhibitory activity was calculated using Formula (2) below.

15 LOX inhibitory activity (%) = 100 − [(A_0_ − A_1_) − (A_2_ − A_1_)] × 100 (2)

where A_0_ is the absorbance of test samples, A_1_ is the absorbance of blanks, and A_2_ is the absorbance of the negative control (DMSO 5%).

The IC_50_ values of tested samples, which represent the concentration leading to 50% inhibition, were determined using the non-linear regression curve of 15-LOX percentage inhibitory activity against the logarithm (log10) of concentrations with GraphPad Prism 6.0 software (GraphPad Software, Inc., San Diego, CA, USA).

### 2.4. Phytochemical Analysis

#### 2.4.1. Total Phenolic Content

The total phenolic content (TPC) of AJHLE was determined using the Folin–Ciocalteu colorimetric method as described by Singleton et al. [31], and adapted to a 96-well microplate by Zhang et al. [32]. In fact, the reaction solution was made by mixing, respectively, 20 µL of extract (5 mg/mL dissolved in ethanol), 100 µL of Folin–Ciocalteu reagent (1 mL of Folin–Ciocalteu reagent in 9 mL of distilled water), and 80 µL of 7.5% (*w/v*) Na_2_CO_3_ solution prepared with deionized water. Thereafter, the reaction solution was incubated in the dark at room temperature (25 °C) for 30 min, and the absorbance of the solution was measured at 765 nm against a blank (ethanol) on a SpectraMax iD3 multi-mode microplate reader (Molecular Devices, USA). The absorbance values were converted to milligrams of gallic acid equivalent (GAE) per gram of dry extract using a standard curve (y = 0.207x + 0.113; R^2^ = 0.997) obtained with a solution of gallic acid (0–100 mg/L).

#### 2.4.2. Total Flavonoid Content

The total flavonoid content (TFC) of AJHLE was determined using the aluminium chloride colorimetric method [33]. The reaction solution was prepared in test tubes by mixing 2 mL of extract (0.3 mg in 1 mL of methanol), 0.1 mL of aluminium chloride hexahydrate solution (10% aqueous AlCl_3_ solution), 0.1 mL of 1M potassium acetate, and 2.8 mL of deionized water. The reaction solution was homogenized and incubated at room temperature (25 °C) for 10 min. Thereafter, 200 µL of each mixture was transferred to a 96-well microplate, and the absorbance was measured at 415 nm on a SpectraMax iD3 multi-mode microplate reader (Molecular Devices, USA). The absorbance values were converted to milligrams of quercetin equivalents (QE) per gram of dry extract using a calibration curve (y = 7.698x + 0.237; R^2^ = 0.999) generated with quercetin (0–0.1 mg/mL).

#### 2.4.3. Liquid Chromatography-Mass Spectrometric (LC-MS) Profiling

Forty milligrams of *A. johnsonii* hydroethanolic leaf extract (AJHLE) was reconstituted in 2 mL of methanol, centrifuged, and then diluted 10-fold with a transfer to glass vials for analysis. A Waters Cyclic Quadrupole time-of-flight (qTOF) mass spectrometer (MS) was used in conjunction with a Waters Acquity ultra-performance liquid chromatograph (UPLC) (Waters, Milford, MA, USA) for high-resolution UPLC-MS analysis. The eluate from the column was initially detected using a Photodiode Array (PDA) detector before entering the mass spectrometer, enabling the simultaneous acquisition of UV and MS spectra. Negative-mode electrospray ionization was used with a cone voltage of 15 V, a desolvation temperature set at 275 °C, a desolvation gas flow rate of 650 L/h, and other MS parameters optimized to achieve the highest resolution and sensitivity. The scanning process involved acquiring data from 150 to 1500 *m*/*z* in both resolution and MSE modes. In the MSE mode, two channels of MS data were obtained: one at a low collision energy of 4 V and the other using a collision energy ramp ranging from 40 to 100 V to capture fragmentation data. To ensure accurate mass determination, leucine enkaphalin was used as a lock mass (reference mass), and the instrument was calibrated with sodium formate. Separation was achieved using a Walters HSS T3, 2.1 × 100 mm, 1.7 μm column. An injection volume of 2 μL was utilized, and the mobile phase consisted of water containing 0.1% formic acid (solvent A) and acetonitrile containing 0.1% formic acid (solvent B). The gradient began at 100% solvent A for 1 min and transitioned linearly to 28% solvent B over 22 min. Subsequently, it progressed to a 40% solvent B within 50 s, followed by a 1.5 min washing step at 100% solvent B. It ended with a re-equilibration to initial conditions for 4 min. The flow rate was set at 0.3 mL/min, while the column temperature was kept constant at 55 °C. A calibration curve established by injecting epigallocatechin gallate (EGCG) (0.5 to 100 mg/L) under the same instrumental conditions was used for the relative quantification of compounds.

The data obtained were processed using MS-DIAL and MS-FINDER (RIKEN Center for Sustainable Resource Science: Metabolome Informatics Research Team, Kanagawa, Japan) after undergoing compression, centroiding, and applying lock-mass correction. [34,35]. Specifically, MS-DIAL was used for the analysis of functions 1 (unfragmented channel) and 2 (fragmented channel) of the Waters MS data, thereby generating MS1 and MS2 spectra, along with extracted ion chromatograms containing the peak height-intensity data. Given that calibration standards are not available for all the detected compounds, the EGCG calibration curve was used in a semi-quantitative manner by interpolating the peak height intensity into concentrations. Each deconvoluted feature (alignment in MS-DIAL), along with its corresponding MS1 and MS2 spectra was transferred from the MS-DIAL to the MS-FINDER for the tentative identification of compounds. Potential compounds were identified based on the accurate mass elemental compositions using available databases and subsequently subjected to in silico fragmentation. By comparing the in silico and measured spectra, a score (out of 10) was assigned to each potential compound match, with the highest score indicating the most probable identification being a minimum score of 4.

### 2.5. Statistical Analysis

All experiments were carried out in triplicate (n = 3) and the results are presented as mean ± SEM (standard error of mean) values. Statistical analysis was carried out with GraphPad Prism 6.0 software (GraphPad Software, Inc., San Diego, CA, USA). The comparison of data among tested samples and/or controls was performed using a one-way analysis of variance (ANOVA), followed by Student–Newman–Keuls or Dunnett’s tests using GraphPad Prism 6.0 software. Results were considered significantly different when the *p* value was less than 0.05.

## 3. Results and Discussion

### 3.1. Antioxidant Activity of A. johnsonii Hydroethanolic Leaf Extract

Three in vitro assays (i.e., ABTS, DPPH, and FRAP) were used to determine the antioxidant activity of AJHLE. These assays are typically based on the scavenging capacity of radicals which are quantified by the color change. The degree of this discoloration or the intensity of the color produced corresponds to the percentage of radicals that have been scavenged. In the current study, AJHLE showed radical scavenging activity in a concentration-dependent manner (Figure S1), and the IC_50_ values are presented in Table 2. It should be noted that a lower IC_50_ value indicates a stronger antioxidant potency. Ascorbic acid and quercetin, known as potent antioxidant compounds and used in our experiments as positive controls, showed the lowest IC_50_ values (4.99 ± 0.20 µg/mL, 4.64 ± 0.85 µg/mL, and 26.68 ± 1.34 µg/mL for ascorbic acid; 2.94 ± 0.46 µg/mL, 3.21 ± 0.19 µg/mL, and 21.62 ± 1.15 µg/mL for quercetin), indicating their highest antioxidant potency against ABTS^•+^, DPPH^•^ and Fe^3+^-TPTZ radicals, respectively. When compared to these known antioxidants, our results showed that AJHLE has strong DPPH and ABTS radical scavenging potency with IC_50_ values of 8.53 ± 1.16 µg/mL and 9.07 ± 1.35 µg/mL, respectively. On the other hand, AJHLE exhibited moderate ferric reducing antioxidant power with an IC_50_ value of 79.09 µg/mL. The DPPH scavenging capacity of the methanolic extract of *A. johnsonii* was reported previously with an IC_50_ value of 1.87 ± 0.08 µg/mL compared to 2.28 ± 0.02 µg/mL for ascorbic acid [21]. The use of different solvents for the extraction of phytochemicals may justify the difference with the DPPH scavenging capacity reported in our study. However, there is no doubt that AJHLE contains antioxidant compounds that should be further chemically characterized. Among the antioxidant compounds from plants, phenolics and flavonoids are the major compounds that are responsible for their antioxidant activity, which intervene via several mechanisms including the regulation of the levels of molecular markers of oxidative stress. In further studies, the effect of AJHLE and its phytoconstituents will be investigated on oxidative-stress markers such glutathione (GSH), catalase, and superoxide dismutase (SOD) to fully elucidate their antioxidative potential.

### 3.2. Anti-Inflammatory Activity of A. johnsonii Hydroethanolic Leaf Extract (AJHLE)

#### 3.2.1. Cytotoxic and Nitric Oxide Inhibitory Effects of AJHLE on LPS-Stimulated RAW 264.7 Macrophages

**Figure 1:**
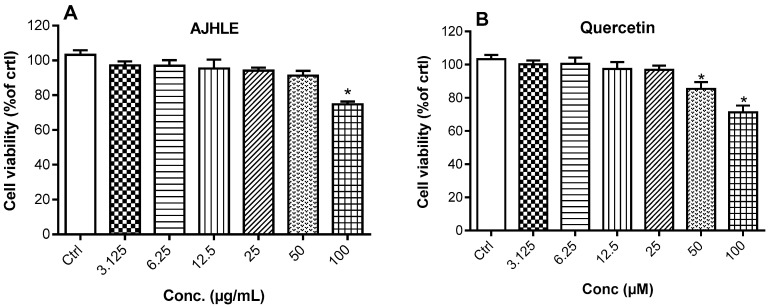
Viability of RAW 264.7 cells treated with different concentrations of *Androstachys johnsonii* hydroethanolic leaf extract (AJHLE) (**A**) and quercetin (**B**). RAW 264.7 cells were treated with increasing concentrations of *A. johnsonii* (3.125–100 µg/mL) and quercetin (3.125–100 µM) for 24 h and the cell viability was evaluated using MTT assay. Data are represented as % of Ctrl (DMSO 0.5%). Each bar represents mean ± SEM of triplicate (n = 3) experiments. Data were analyzed by one-way ANOVA followed by Dunnett’s test. * *p* < 0.05 vs. Ctrl.

To determine the nitric oxide (NO) inhibitory effect of AJHLE, we initially determine its potential cytotoxic effect on RAW 264.7 macrophages. In this regard, the cells were treated with increasing concentrations (3.125–100 µg/mL) of AJHLE for 24 h, and the cell viability was measured using the MTT assay, a technique which evaluates the ability of viable cells to reduce intracellular MTT into formazan crystals via the oxidoreductase and dehydrogenase enzymes of the electron transport chains [27,36]. First, cells treated with DMSO (0.5%) were used as controls and set as 100% cell viability, and the results of cells treated with tested samples are presented as a percentage of the control. AJHLE was found to be non-cytotoxic at up to 50 µg/mL but showed significant (*p* < 0.05) cytotoxic effects at 100 µg/mL (≈80% cell viability) compared to the control (DMSO 0.5%) (Figure 1A). Quercetin, used as a positive control in the anti-inflammatory assay, was also found to significantly reduce cell viability at 50 and 100 µM (Figure 1B). Therefore, only non-cytotoxic concentrations were used in subsequent experiments involving RAW 264.7 macrophages. The selection of these non-toxic concentrations is mainly because the decrease in NO level, or the regulation of the expression of oxidative-stress molecular markers released by cells should not be attributed to the reduction of the cell viability.

**Figure 2:**
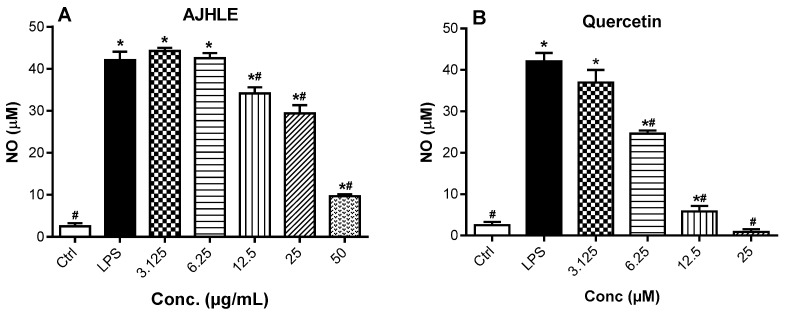
Nitric oxide (NO) production in LPS-stimulated RAW 264.7 cells pre-treated with different concentrations of *Androstachys johnsonii* hydroethanolic leaf extract (AJHLE) (**A**) and quercetin (**B**). RAW 264.7 cells were pre-treated with increasing concentrations of *A. johnsonii* (3.125–100 µg/mL) and quercetin (3.125–100 µM) for 2 h, followed by exposure to 1 µg/mL of LPS for 24 h. Each bar represents means ± SEM of triplicate (n = 3) experiments. The data were analyzed using one-way ANOVA followed by Student–Newman–Keuls or Dunnett’s tests. * *p* < 0.05 vs. Ctrl. # *p* < 0.05 vs. LPS.

One of the assays used to determine the anti-inflammatory activity of AJHLE was to measure NO production in culture supernatants of LPS-stimulated RAW 264.7 macrophages using the Griess reagent. NO is a signaling molecule produced by inducible nitric oxide synthase (iNOS) and is a recognized mediator and regulator of inflammatory responses [37,38]. An excessive production of NO can induce oxidative stress, thereby causing damaging effects to the cells. Therefore, the inhibition of NO production is an effective way to control inflammatory responses and oxidative stress. As presented in Figure 2, the LPS treatment of RAW 264.7 macrophages induced a significant NO production compared to untreated cells (Ctrl). Moreover, AJHLE as well as quercetin prevented LPS-induced NO production in a concentration-dependent manner with IC_50_ values of 24.06 ± 1.34 µg/mL and 7.08 ± 1.01 µM, respectively (Figure S2). These results showed that AJHLE exerts its anti-inflammatory effect by preventing NO release in LPS-stimulated RAW 264.7 macrophages. As mentioned above, these results suggested that AJHLE contains antioxidant compounds which may be involved in the decrease in nitric oxide levels. Since the release of NO and cytokines in LPS-stimulated cells is activated by ROS production, further investigation on the mechanism of action of AJHLE is needed on other inflammatory mediators to support its efficacy for the management of oxidative stress and inflammatory conditions.

#### 3.2.2. Regulatory Effect of AJHLE on the Expression of Pro-and Anti-Inflammatory Mediators

To further validate the anti-inflammatory effect of AJHLE, we investigated its potential to regulate the expression of pro-inflammatory mediators (IL-1β, TNF-α, and COX-2) and anti-inflammatory cytokine (IL-10) in LPS-stimulated RAW 264.7 macrophages. COX-2, also named prostaglandin-endoperoxide synthase 2, is an inducible enzyme expressed in response to inflammatory stimuli, and which plays a pivotal role in the production of prostaglandins that support the inflammatory process [39,40]. Additionally, pro-inflammatory cytokines (IL-1β, TNF-α) are responsible for initiating inflammation while the anti-inflammatory cytokine (IL-10) is released to stop or lessen excessive inflammatory reactions [41,42]. Therefore, targeting the expression of COX-2 and the above-mentioned cytokines is an effective approach to regulate both acute and chronic inflammation. For this purpose, cells were pre-treated with non-cytotoxic concentrations of AJHLE and quercetin for 2 h, followed by LPS exposure for another 24 h. Thereafter, cell lysates were used to evaluate the expression of inflammatory mediators such as COX-2, IL-1β, IL-10, and TNF-α using commercially available kits. Quercetin was used as a positive control as it exerts its anti-inflammatory property by regulating the release of inflammatory cytokines and enzymes, making it a potential therapeutic agent for various inflammatory conditions [43].

**Figure 3:**
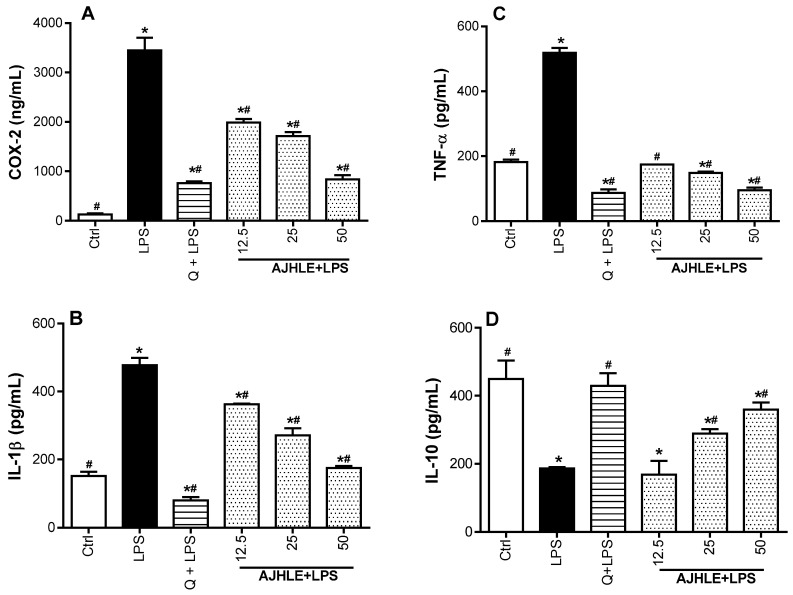
Regulatory effect of *Androstachys johnsonii* hydroethanolic leaf extract (AJHLE) on the expression of COX-2 (**A**), IL-1β (**B**), TNF-α (**C**), and IL-10 (**D**). RAW 264.7 cells were pre-treated with different concentrations (12.5, 25 and 50 µg/mL) of AJHLE and quercetin (Q) at 25 µM for 2 h, followed by exposure to 200 ng/mL of LPS for 24 h. Each bar represents means ± SEM of duplicate (n = 2) experiments. The data were analyzed using one-way ANOVA followed by Student–Newman–Keuls or Dunnett’s tests. * *p* < 0.05 vs. Ctrl. # *p* < 0.05 vs. LPS.

As indicated in Figure 3, RAW 264.7 cells exposed to LPS significantly (*p* < 0.05) induced the expression of pro-inflammatory mediators (IL-1β, TNF-α, and COX-2), and suppressed the release of anti-inflammatory cytokine(IL-10), with respect to control cells. The treatment of RAW 264.7 cells with AJHLE significantly (*p* < 0.05) downregulated the expression of all pro-inflammatory mediators (IL-1β, TNF-α, and COX-2) and upregulated the expression of cytokine (IL-10) compared to LPS-treated cells. Interestingly, the expression of TNF-α and COX-2 was efficiently downregulated after treatment with AJHLE (50 µg/mL) compared with quercetin (25 µM). These results demonstrated that AJHLE has strong anti-inflammatory activity and counteracts effectively the LPS-stimulated inflammatory status in RAW 264.7 cells via the regulation of the expression of inflammatory mediators. Taken together, the combined inhibitory effect of AJHLE on NO production and pro-inflammatory cytokines can be considered a promising step towards the validation of *A. johnsonii* as a potential source of anti-inflammatory agents.

#### 3.2.3. Protective Effect of AJHLE on Reactive Oxygen Species Production

Long-term inflammation process increases reactive oxygen species (ROS) production, causing oxidative stress. ROS are metabolic products arising from various cells, and which play an essential role in the initiation and/or progression of inflammatory disorders via the activation of oxidative-stress signaling pathways [44,45]. The regulation of oxidative stress induced by excessive ROS production can be controlled either by the antioxidant defense systems or by the supplementation of natural antioxidants from food and medicinal plants [45]. Therefore, we examined the potential protective effect of AJHLE on LPS-stimulated oxidative stress on RAW 264.7 macrophages by measuring the levels of intracellular ROS, using DCFH-DA as a probe.

**Figure 4:**
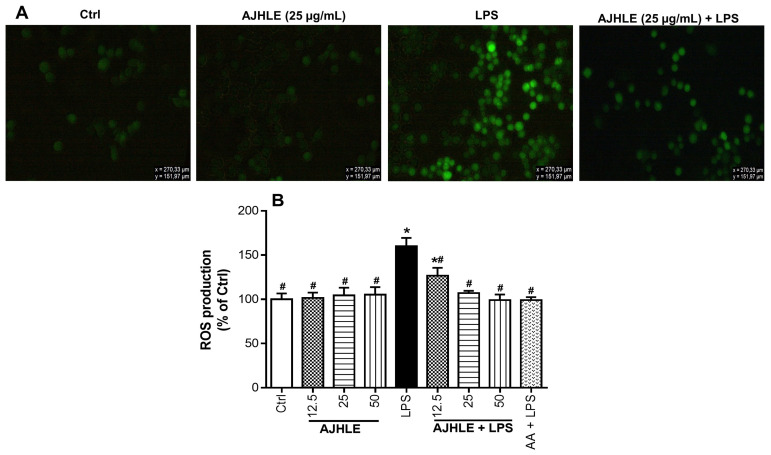
Protective effect of *Androstachys johnsonii* hydroethanolic leaf extract (AJHLE) on reactive oxygen species (ROS) production in LPS-stimulated RAW 264.7 cells. RAW 264.7 cells were pre-treated with different concentrations (12.5, 25 and 50 µg/mL) of AJHLE and ascorbic acid (AA) at 25 µg/mL for 2 h, followed by exposure to 200 ng/mL of LPS for 24 h. Intracellular ROS levels were measured using the probe DCFH-DA (10 µM), and the cell fluorescence was measured at 485 nm (excitation) and 535 nm (emission) (**A**). Intracellular ROS levels were expressed as percentages of negative control cells (**B**). Each bar represents the mean ± SEM of triplicate (n = 3) experiments. Data were analyzed by one-way ANOVA followed by Student–Newman–Keuls or Dunnett’s tests. * *p* < 0.05 vs. Crtl; # *p* < 0.05 vs. LPS.

As observed in Figure 4A, the treatment of RAW 264.7 macrophages with AJHLE did not cause a significant production of ROS compared to control cells (Ctrl), while the exposure of RAW 264.7 macrophages to LPS provoked a significant increase in ROS production compared to control cells (Ctrl). Interestingly, the pre-treatment of RAW 264.7 cells with the extract (AJHLE+LPS) significantly (*p* < 0.05) prevented LPS-stimulated ROS production. Additionally, the percentage of ROS-production inhibition via AJHLE was found to decrease in a concentration-dependent manner (Figure 4B) with an IC_50_ value of 10.49 ± 1.19 µg/mL (Table 3). Ascorbic acid, a known antioxidant and anti-inflammatory molecule which regulates the level of ROS at early stages of their formation [46], was found to strongly prevent ROS production after LPS exposure. ROS produced by activated macrophages are responsible for the inflammation injury by stimulating the release of inflammatory mediators such as NO and cytokines. In this manner, the preventive effect of AJHLE on ROS production is a clear indication of its potential to inhibit the release of NO and regulate the expression of inflammatory mediators, as observed above. Taken together, these results support the protection of AJHLE against the deleterious effect of ROS in LPS-stimulated RAW 264.7 macrophages, thereby suggesting the use of this plant extract for the prevention of inflammatory and oxidative-stress-related disorders.

#### 3.2.4. 15-Lipoxygenase Inhibitory Activity of AJHLE

Another assay used to prove the anti-inflammatory activity of AJHLE was to evaluate the 15-lipoxygenase (15-LOX)-inhibitory activity of AJHLE. 15-LOX regulates the inflammatory responses via the generation of pro-inflammatory lipid mediators known as leukotrienes, and several studies have shown its involvement in the pathogenesis of a variety of human diseases [47]. The inhibition of 15-LOX can be used as a therapeutic approach to reduce the generation of lipid mediators and stop inflammation. Therefore, the ferrous oxidation-xylenol orange (FOX) assay was used to determine the 15-lipoxygenase inhibitory activity of AJHLE. The results showed that AJHLE inhibits 15-LOX activity in a concentration-dependent manner (Figure S3) with an IC_50_ value of 37.71 ± 1.03 µg/mL. Gallic acid, a potent 15-LOX inhibitor, showed an IC_50_ value of 22.08 ± 1.96 µg/mL, which is significantly (*p* < 0.05) lower than that of AJHLE. Despite this, AJHLE can be considered a potential source of 15-LOX inhibitors. Therefore, the isolation and identification of compounds responsible for the efficacy of AJHLE becomes important, as this may lead to the discovery of novel therapeutic agents against inflammatory-related diseases.

### 3.3. Phytochemical Composition of AJHLE

**Figure 5:**
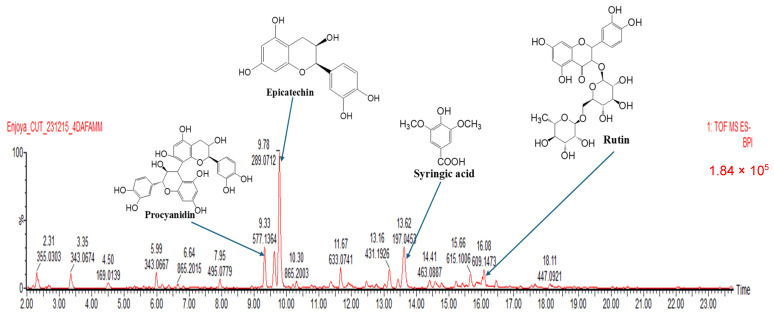
Liquid chromatography-mass spectrometric (LC-MS) profile of *A. johnsonii* hydroethanolic leaf extract (AJHLE). Two microliters of AJHLE were injected to Waters Cyclic Quadrupole time-of-flight (qTOF) mass spectrometer (MS) connected to a Waters Acquity ultra-performance liquid chromatograph (UPLC) (Waters, Milford, MA, USA) for high-resolution UPLC-MS analysis. Column eluate first passed through a Photodiode Array (PDA) detector before going to the mass spectrometer, allowing simultaneous collection of UV and MS spectra. Major compounds detected were procyanidin (Rt: 9.33 min), epicatechin (Rt: 9.78 min), syringic acid (Rt: 13.62 min), and rutin (Rt: 16.08 min).

Flavonoids and phenolics are well known as important natural bioactive compounds with potential benefits for human health, especially for their usage in the prevention and treatment of various diseases. These two groups of compounds play a major role in protecting biological systems against harmful effects of oxidative stress, and they have been reported to exert several biological properties including antioxidant and anti-inflammatory activities [48]. Due to their importance in human health, we decided to quantify their concentrations in *A. johnsonii* hydroethanolic leaf extract (AJHLE). As presented in Table 1, AJHLE was found to be very rich in flavonoids and phenolic compounds with 33.57 ± 1.40 mgQE/g of dry extract and 161.27 ± 0.90 mgGAE/g of dry extract, respectively. Flavonoids and phenolic compounds are recognized as antioxidant and anti-inflammatory agents [49,50,51], so the high contents of AJHLE with these phytochemicals suggest their implication in the antioxidant and anti-inflammatory activities of *A. johnsonii*. Moreover, the LC-MS profile of AJHLE also confirmed the presence flavonoids and phenolic compounds as well as other classes of compounds such as hydrolysable tannins, quinic acid derivatives, and lignan glycosides (Table 4), and the major compounds identified were procyanidin (RT: 9.33 min), epicatechin (RT: 9.78 min), syringic acid (RT: 13.62 min), and rutin (RT: 16.08 min) (Figure 5).

The most abundant compound within AJHLE was epicatechin (6193 mg/L), which is a flavonoid present in different fruits and tea leaves. Many in vitro and in vivo studies support the antioxidant and anti-inflammatory activities of epicatechin by inhibiting the production of NO, PGE_2_, and proinflammatory cytokines (IL-1β, IL-6, and TNF-α) as well as downregulating the expression of iNOS and COX-2 in LPS-induced RAW 264.7 cells which occur through the inactivation of NF-κB signaling pathways [52,53,54,55]. Similarly, high concentrations of rutin (1158 mg/L) and procyanidin (1867 mg/L) were found in AJHLE, and several studies have confirmed their potent antioxidant and anti-inflammatory activities, which function via their protective mechanism against oxidative stress caused by ROS production [56,57,58], and their capability to reduce the excessive expression of inflammatory mediators such as IL-6, TNF-α, PGE_2_, NO, and COX-2 through the regulation of nuclear factor erythroid 2-related factor 2 (Nrf2), MAPK, and NF-κB pathways [59,60,61,62,63]. In addition, syringic acid (1971 mg/L), a natural phenolic acid found in vegetables, fruits, and other plant-based foods, was also detected in AJHLE. The antioxidant and anti-inflammatory effects of syringic acid has been demonstrated via the reduction of oxidative-stress markers (HOO•, HO•, TBARS, and NO_2_) [64,65] and the downregulation of iNOS and COX-2 expression as well as the modulation of NF-κB and the Janus kinase-signal transducer of activation (JAK-STAT) signaling pathways [66,67]. Overall, the detection of these major compounds within AJHLE may justify its strong antioxidant and anti-inflammatory activities, therefore indicating its potential as a source of therapeutic agents against inflammatory and oxidative-stress-related conditions. However, several unknown compounds are still not yet identified, thus opening further perspectives towards the isolation of bioactive compounds in *A. johnsonii*, which remains to date an under-explored plant species.

## 4. Conclusions

In summary, data obtained from the present study demonstrated that *A. johnsonii* hydroethanolic leaf extract (AJHLE) has the capacity to scavenge free radicals such as ABTS^•+^, DPPH^•^, and Fe^3+^-TPTZ, and it also strongly suppresses the production of pro-inflammatory mediators such as NO, IL-1β, TNF-α, and COX-2 in LPS-stimulated RAW 264.7 macrophages. Additionally, AJHLE was able to inhibit the 15-LOX enzymatic activity, upregulates the release of IL-10 cytokine, and provides protection against ROS production. The reported antioxidant and anti-inflammatory activities of AJHLE could be associated with its phytochemical contents where consistent amounts of phenolics and flavonoids were quantified together with significant concentrations of compounds such as procyanidin, epicatechin, rutin, and syringic acid. These results agree with the available literature, which emphasizes the correlation between the antioxidant and anti-inflammatory potentials and the presence of identified phytochemicals. These results open the step towards further pharmacological and phytochemical studies aimed at isolating and characterizing bioactive compounds for the development of new therapeutic agents against inflammatory and oxidative-stress-related conditions.

## Figures and Tables

**Table 1:** Yield of extraction and phytochemical contents of *A. johnsonii* hydroethanolic leaf extract (AJHLE).

	Yield of Extraction (g Extract/100 g Dry Material)	Phenolic Content (mgGAE/g Extract)	Flavonoid Content (mgQE/g Extract)
AJHLE	16.23	161.27 ± 0.90	33.57 ± 1.40

Data are presented as means of triplicate measurements ± standard error of mean (SEM). GAE: gallic acid equivalent; QE: quercetin equivalent.

**Table 2:** In vitro antioxidant activity of *A. johnsonii* hydroethanolic leaf extract (AJHLE).

IC_50_ (µg/mL)			
Sample	ABTS	DPPH	FRAP
AJHLE	9.07 ± 1.35	8.53 ± 1.16	79.09 ± 1.16
Ascorbic acid	4.99 ± 0.20 *	4.64 ± 0.85 *	26.68 ± 1.34 *
Quercetin	2.94 ± 0.46 *	3.21 ± 0.19 *	21.62 ± 1.15 *

Data are presented as means of triplicate measurements ± standard error of mean (SEM); IC_50_: concentration required to scavenge 50% of each radical as compared to negative control. (*) means significant difference (*p* < 0.05).

**Table 3:** Anti-inflammatory potential of *A. johnsonii* hydroethanolic leaf extract (AJHLE).

	IC_50_ (µg/mL)		
Sample	NO	15-LOX	ROS
AJHLE	24.06 ± 1.34	37.71 ± 1.03	10.49 ± 1.19
Gallic acid	-	22.08 ± 1.96 *	-
Quercetin (µM)	7.08 ± 1.01	-	-

Data are presented as means of triplicate measurements ± standard error of mean (SEM); IC_50_: concentration required to inhibit the biological effect by 50% compared to the negative control. (*) means significant difference (*p* < 0.05). (-): not determined.

**Table 4:** Phytochemical compounds detected and characterized in *A. johnsonii* hydroethanolic leaf extract, using liquid chromatography-mass spectrometric equipment in negative-mode ionization.

Peak N^o^	RT (min)	[M-H]-(*m*/*z*)	Tentative Assignment (Compound Name)	Ontology	Molecular Formula	Total Score	Peak Height Intensity	Conc. in Extract vs. EGCG (mg/L)
1	5.99	343.06	Theogallin	Quinic acids and derivatives	C_14_H_16_O_10_	7.27	16,943	626
2	6.64	865.19	Procyanidin C1	Biflavonoids and polyflavonoids	C_45_H_38_O_18_	7.49	6920	256
3	7.95	495.07	Hibiscitrin	Flavonoid-3-O-glycosides	C_21_H_20_O_14_	7.10	10,731	397
4	9.33	577.13	**Procyanidin**	Biflavonoids and polyflavonoids	C_30_H_26_O_12_	6.12	50,504	**1867**
5	9.73	311.04	Caftaric acid	Coumaric acids and derivatives	C_13_H_12_O_9_	4.57	3,979	147
6	9.78	289.07	**Epicatechin**	Catechins	C_15_H_14_O_6_	6.79	167,515	**6193**
7	11.36	541.05	Putranjivain A	Hydrolyzable tannins	C_46_H_36_O_31_	4.38	8535	316
8	11.67	633.07	Corilagin	Hydrolyzable tannins	C_27_H_22_O_18_	7.19	24,505	906
9	12.46	729.14	ent-epicatechin 3-gallate	Biflavonoids and polyflavonoids	C_37_H_30_O_16_	5.06	7375	273
10	12.77	631.09	8-C-Ascorbylepigallocatechin 3-gallate	Catechin gallates	C_28_H_24_O_17_	4.54	4531	168
11	13.03	479.08	Myricetin 3-galactoside	Flavonoid-3-O-glycosides	C_21_H_20_O_13_	6.84	4306	159
12	13.43	577.13	Procyanidin B5	Biflavonoids and polyflavonoids	C_30_H_26_O_12_	7.19	11,537	427
13	13.62	197.04	**Syringic acid**	Gallic acid and derivatives	C_9_H_10_O_5_	6.75	53,310	**1971**
14	14.41	463.08	Quercetin 3-galactoside	Flavonoid-3-O-glycosides	C_21_H_20_O_12_	8.11	9361	346
15	14.51	625.14	Quercetin 3-glucosyl-2-galactoside	Flavonoid-3-O-glycosides	C_27_H_29_O_17_	7.22	1743	64
16	14.58	953.09	Chebulagic acid	Hydrolyzable tannins	C_41_H_30_O_27_	4.89	6614	245
17	14.78	481.09	3′-(2″-Galloylglucosyl)-phloroacetophenone	Phenolic glycosides	C_21_H_22_O_13_	5.46	4214	156
18	15.23	595.13	Quercetin 3-lathyroside	Flavonoid-3-O-glycosides	C_26_H_28_O_16_	6.68	8662	320
19	15.47	581.22	(7′R)-(+)-Lyoniresinol 9′-glucoside	Lignan glycosides	C_28_H_38_O_13_	6.25	755	28
20	15.66	615.10	Quercetin 3-(2-galloylglucoside)	Flavonoid-3-O-glycosides	C_28_H_24_O_16_	5.09	6334	234
21	16.08	609.14	**Rutin**	Flavonoid-3-O-glycosides	C_27_H_30_O_16_	8.09	31,331	**1158**
22	16.14	463.08	Kaempferol 3-alpha-l-arabinopyranoside	Flavonoid-3-O-glycosides	C_20_H_18_O_10_	6.13	1380	51
23	16.88	447.09	Quercitrin	Flavonoid-3-O-glycosides	C_21_H_20_O_11_	8.09	2532	94
24	17.55	505.20	Icariside E4	2-arylbenzofuran flavonoids	C_26_H_34_O_10_	5.89	3333	123
25	17.59	515.12	3,5-Dicaffeoylquinic acid	Quinic acids and derivatives	C_25_H_24_O_12_	6.11	467	17
26	17.65	599.10	Kaempferol 7-(6″-galloylglucoside)	Flavonoid-7-O-glycosides	C_28_H_24_O_15_	5.43	4681	173
27	17.72	593.15	Astragalin 7-rhamnoside	Flavonoid-7-O-glycosides	C_27_H_30_O_15_	8.12	2021	75
28	18.11	447.09	Astragalin	Flavonoid-7-O-glycosides	C_21_H_20_O_11_	8.09	4786	177

RT: retention time; EGCG: epigallocatechin gallate. Bold indicates compounds detected in significant amounts in the extract.

## Data Availability

The datasets used and/or analyzed during the current study are available from the corresponding authors on reasonable request.

## Supplementary Materials

The following supporting information can be downloaded at: https://www.mdpi.com/article/10.3390/antiox13040401/s1. Figure S1: Non-linear regression curves for IC_50_ determination of *Androstachys johnsonii* hydroethanolic leaf extract (AJHLE) in *ABTS* (**A**), DPPH (**B**), and FRAP (**C**) assays. Figure S2: Non-linear regression curves for IC_50_ determination of *Androstachys johnsonii* hydroethanolic leaf extract (AJHLE) (**A**) and quercetin (**B**) in nitric oxide (NO)-production-inhibitory assay. Figure S3: Non-linear regression curves for IC_50_ determination of *Androstachys johnsonii* hydroethanolic leaf extract (AJHLE) (**A**) and gallic acid (**B**) in 15-lipoxygenase (15-LOX)-inhibitory assay.
