# Supplementary material for: Phytoconstituents of Androstachys johnsonii Prain Prevent Reactive Oxygen Species Production and Regulate the Expression of Inflammatory Mediators in LPS-Stimulated RAW 264.7 Macrophages
Source: Antioxidants (Basel). 2024 Mar 27;13(4):401. doi: 10.3390/antiox13040401 (PMC11047428; doi:10.3390/antiox13040401)
Supplement: Supplementary file 1 [file antioxidants-13-00401-s001.zip › antioxidants-2903860-supplementary.pdf]

Supplementary material

Phytoconstituents of *Androstachys johnsonii* Prain Prevent Reactive Oxygen Species Production and Regulate the Expression of Inflammatory Mediators in LPS-Stimulated RAW 264.7 Macrophages

Emmanuel Mfotie Njoya *, Gaetan T. Tabakam, Chika I. Chukwuma, Samson S. Mashele and Tshepiso J. Makhafola *

Centre for Quality of Health and Living, Faculty of Health and Environmental Sciences, Central University of Technology, Bloemfontein 9300, Free State, South Africa; tgaetan@cut.ac.za (G.T.T.);
cchukwuma@cut.ac.za (C.I.C.); smashele@cut.ac.za (S.S.M.)

* Correspondence: mfotiefr@yahoo.fr or enjoya@cut.ac.za (E.M.N.); jmakhafola@cut.ac.za (T.J.M.)

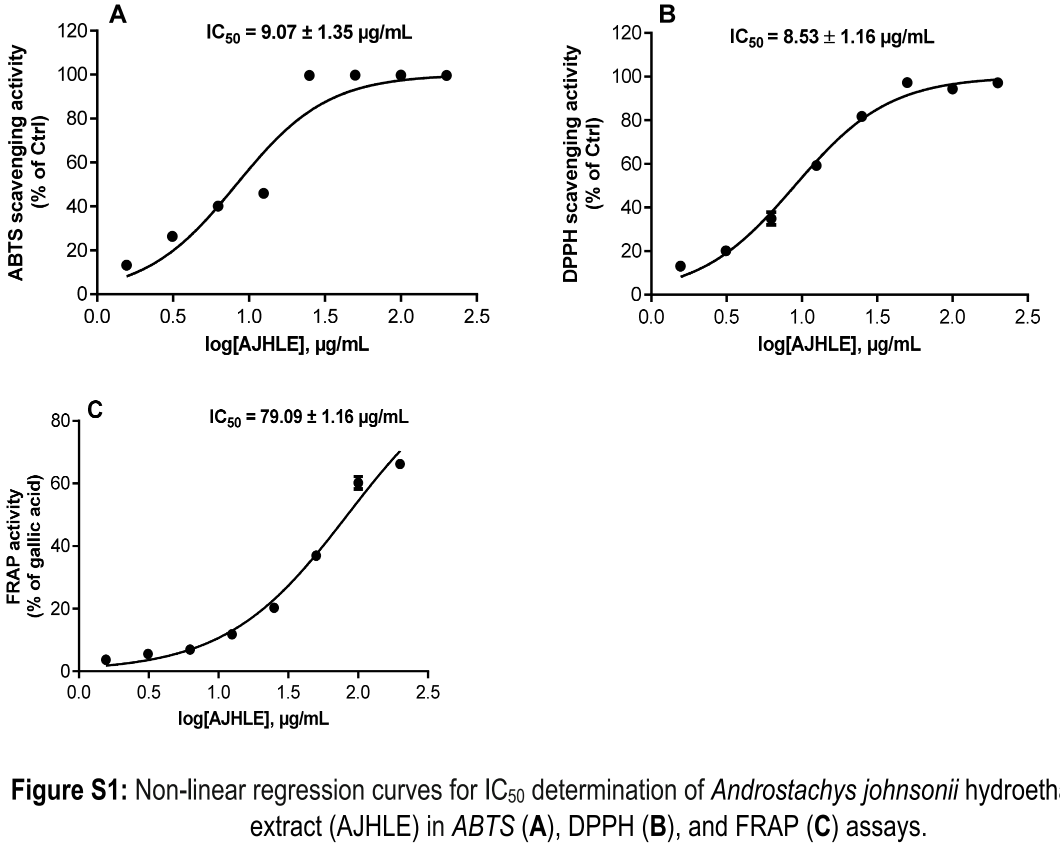

Figure S1: Non-linear regression curves for IC_{50} determination of *Androstachys johnsonii* hydroethanolic leaf extract (AJHLE) in *ABTS* (**A**), DPPH (**B**), and FRAP (**C**) assays.

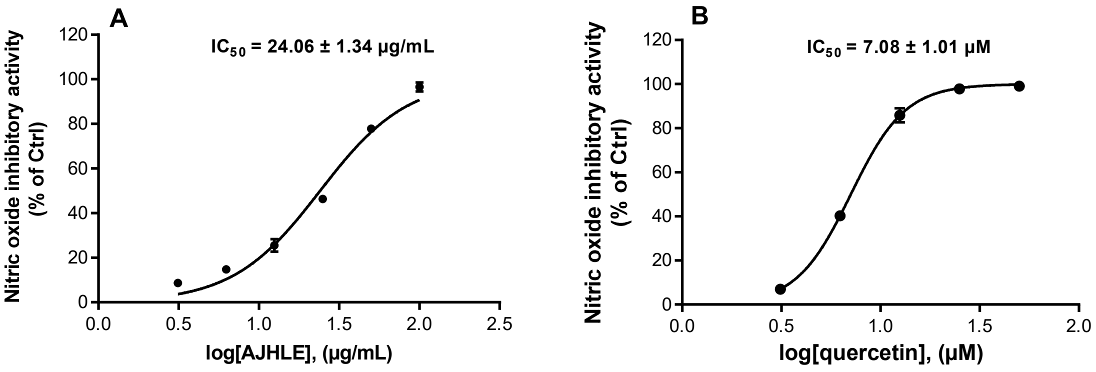

Figure S2: Non-linear regression curves for IC_{50} determination of *Androstachys johnsonii* hydroethanolic leaf extract (AJHLE) (**A**) and quercetin (**B**) in *nitric oxide (NO)* production inhibitory assay.

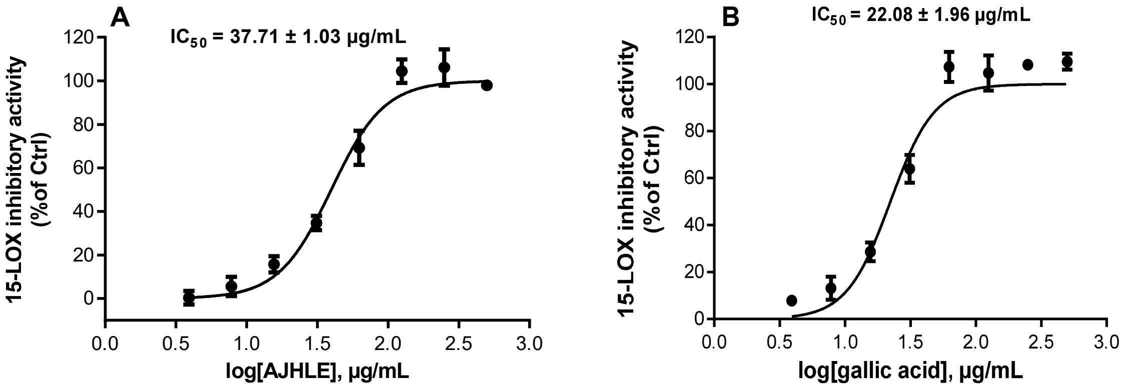

Figure S3: Non-linear regression curves for IC_{50} determination of *Androstachys johnsonii* hydroethanolic leaf extract (AJHLE) (**A**) and gallic acid (**B**) in 15-lipoxygenase (15-LOX) inhibitory assay.
